# HIV Viral Load Monitoring Among Patients Receiving Antiretroviral Therapy — Eight Sub-Saharan Africa Countries, 2013–2018

**DOI:** 10.15585/mmwr.mm7021a2

**Published:** 2021-05-28

**Authors:** Shirley Lee Lecher, Peter Fonjungo, Dennis Ellenberger, Christiane Adje Toure, George Alemnji, Nancy Bowen, Frank Basiye, Anita Beukes, Sergio Carmona, Michael de Klerk, Karidia Diallo, Eric Dziuban, Charles Kiyaga, Henry Mbah, Johannes Mengistu, Tsietso Mots’oane, Christina Mwangi, Jane W. Mwangi, Michael Mwasekaga, Jonathan N’tale, Mary Naluguza, Isaac Ssewanyana, Wendy Stevens, Innocent Zungu, Ravikiran Bhairavabhotla, Helen Chun, Nicholas Gaffga, Stephen Jadczak, Spencer Lloyd, Shon Nguyen, Ritu Pati, Katrina Sleeman, Clement Zeh, Guoqing Zhang, Heather Alexander

**Affiliations:** ^1^Division of Global HIV and TB, Center for Global Health, CDC; ^2^Division of Global HIV and TB, Center for Global Health, CDC Country Office, Abidjan, Côte d’Ivoire; ^3^Office of the Global AIDS Coordinator, U.S. Department of State, Washington, DC; ^4^Ministry of Health, Nairobi, Kenya; ^5^Division of Global HIV and TB, Center for Global Health, CDC Country Office, Nairobi, Kenya; ^6^Division of Global HIV and TB, Center for Global Health, CDC Country Office, Windhoek, Namibia; ^7^Department of Molecular Medicine and of Haematology, National Health Laboratory Service, Johannesburg, South Africa; ^8^Division of Global HIV and TB, Center for Global Health, CDC Country Office, Pretoria, South Africa; ^9^Central Public Health Laboratory, Kampala Uganda; ^10^Division of Global HIV and TB, Center for Global Health, CDC Country Office, Lilongwe, Malawi; ^11^Division of Global HIV and TB, Center for Global Health, CDC Country Office, Maseru, Lesotho; ^12^Ministry of Health, Maseru, Lesotho; ^13^Division of Global HIV and TB, Center for Global Health, CDC Country Office, Kampala, Uganda; ^14^Division of Global HIV and TB, Center for Global Health, CDC Country Office, Dar es Salaam, Tanzania.

One component of the Joint United Nations Programme on HIV/AIDS (UNAIDS) goal to end the HIV/AIDS epidemic by 2030, is that 95% of all persons receiving antiretroviral therapy (ART) achieve viral suppression.^†^ Thus, testing all HIV-positive persons for viral load (number of copies of viral RNA per mL) is a global health priority (*1*). CDC and other U.S. government agencies, as part of the U.S. President’s Emergency Plan for AIDS Relief (PEPFAR), together with other stakeholders, have provided technical assistance and supported the cost for multiple countries in sub-Saharan Africa to expand viral load testing as the preferred monitoring strategy for clinical response to ART. The individual and population-level benefits of ART are well understood (*2*). Persons receiving ART who achieve and sustain an undetectable viral load do not transmit HIV to their sex partners, thereby disrupting onward transmission (*2*,*3*). Viral load testing is a cost-effective and sustainable programmatic approach for monitoring treatment success, allowing reduced frequency of health care visits for patients who are virally suppressed (*4*). Viral load monitoring enables early and accurate detection of treatment failure before immunologic decline. This report describes progress on the scale-up of viral load testing in eight sub-Saharan African countries from 2013 to 2018 and examines the trajectory of improvement with viral load testing scale-up that has paralleled government commitments, sustained technical assistance, and financial resources from international donors. Viral load testing in low- and middle-income countries enables monitoring of viral load suppression at the individual and population level, which is necessary to achieve global epidemic control. Although there has been substantial achievement in improving viral load coverage for all patients receiving ART, continued engagement is needed to reach global targets.

Scale-up of HIV viral load testing has been a global priority following release of the World Health Organization (WHO) 2013 ART guidelines, which recommended using viral load instead of CD4 counts as the preferred approach to monitoring ART effectiveness (*5*). In 2016, the ART guidelines were revised to recommend viral load testing (rather than CD4 cell counts) for all HIV-positive persons to monitor effectiveness (*1*). These guidelines promote the UNAIDS goal to end the HIV/AIDS epidemic by 2030, with 95% of patients receiving ART having viral suppression by 2030 (*1*). Global ART expansion has increased demand for viral load monitoring. In 2018, 23.3 million persons were receiving ART, an increase of nearly 200%, compared with 8 million in 2010 (*6*). Country viral load testing capacity continues to grow. For example, the total number of health facilities in Kenya offering viral load testing increased approximately 180%, from 722 (in 218 districts) in 2012 to approximately 2,000 (in approximately 300 districts) in 2016 (*7*).

Globally, approximately two thirds of the HIV-infected persons reside in Africa.^§^ To evaluate progress in scale-up of HIV viral load testing, investigators assessed activities and expansion in eight sub-Saharan African countries (Côte d’Ivoire, Kenya, Lesotho, Malawi, Namibia, South Africa, Tanzania, and Uganda) during 2013–2018. Data from an earlier assessment of annual progress of viral load scale-up for all the countries except Lesotho were published in 2015 and 2016 (*8*,*9*). For this assessment, the questionnaire used for the previous reports was updated to obtain annual data for Lesotho from 2013 through 2018 and data from 2016 through 2018 for all other countries. Countries were selected based on availability of data and agreement with their ministries of health. Data were collected for each calendar year. Country guidelines called for viral load testing at 6 months after ART initiation, followed by testing at 12 months and annually thereafter (except Malawi, which recommended viral load testing every 2 years). Ministry of health officials and CDC program officers jointly collected information from the laboratory information system on the cumulative number of ART patients, the number of ART patients with at least one viral load test result, the percentage of viral load tests results showing viral suppression (defined as ≤1,000 HIV RNA copies per mL), and the mean turnaround time from sample collection to release of viral load test results.

As of early 2019, South Africa had the largest number of patients receiving ART (4.57 million) among all countries studied (Table), representing approximately 59% of persons in South Africa living with HIV based on UNAIDS estimates (*10*). From 2013 to 2018, the total number of patients receiving ART increased by 78% across all eight countries, from 5,190,275 before scale-up to 9,240,111 in 2018, increasing the demand for viral load testing. During this period, the average turnaround time from sample collection to release of test results decreased in Kenya (55.6%), Lesotho (50%), and Uganda (22.2%). However, turnaround time increased in Côte d’Ivoire, Namibia, South Africa, and Tanzania; the turnaround time in Malawi did not change.

**TABLE:** Selected indicators for viral load monitoring before and after scale-up*^,†^ of viral load testing, by country — eight sub-Saharan African countries, 2013–2014 and 2018

Country	Cumulative no. of patients^§^ receiving ART		Avg. interval from sample collection to return of VL test results to referring facility, days		% of ART VL tests indicating viral suppression	
	Before scale-up^†^	2018 (% change)	Before scale-up^†^	2018 (% change)	Before scale-up^†^	2018 (% change)
Côte d'Ivoire	129,993	248,194 (91)	10	15 (50)	66	78 (18)
Kenya	631,503	1,069,451 (69)	18	8 (–56)	64	90 (41)
Lesotho	111,322	218,493 (96)	56	28 (–50)	75	93 (24)
Malawi	472,865	805,323 (70)	18	18 (0)	86	86 (0)
Namibia	126,779	180,584 (42)	5	6 (20)	74	94 (28)
South Africa^¶^	2,609,275	4,551,331 (74)	3	4 (33)	75	85 (13)
Tanzania^¶^	600,886	999,628 (66)	10	27 (170)	80	85 (6)
Uganda	507,663	1,167,107 (130)	18	14 (–22)	90	88 (–2)
**Total**	**5,190,275**	**9,240,111 (78)**	**—**	**—**	**—**	**—**

**Abbreviations:** ART = antiretroviral therapy; VL = viral load; WHO = World Health Organization.

* Scale-up refers to the beginning of monitoring patients on ART with HIV viral load testing rather than CD4 cell testing as recommended in WHO guidelines as the preferred monitoring strategy. Because countries were not monitoring HIV patients with viral load testing, it was necessary to start viral load testing and scale-up to test all patients on ART.

^†^ Period before scale-up was 2014 in Côte d’Ivoire and 2013 in all other countries.

^§^ Adult and pediatric patients.

^¶^ South Africa and Tanzania reported through June 2018.

During 2013–2018, the proportion of ART patients who had at least one viral load test result increased 1,850% in Côte d’Ivoire (from 3.8% to 74.1%), 921% in Kenya (from 8.4% to 85.8%), 959% in Lesotho (from 4.9% to 51.9%), 755% in Malawi (from 6% to 51.3%), 65% in Namibia (from 60.5% to 99.9%), and 1,716% in Uganda (from 4.9% to 89%) (Figure 1). South Africa and Tanzania were excluded from this analysis because 2018 data were only available for January through June.

**FIGURE 1:**
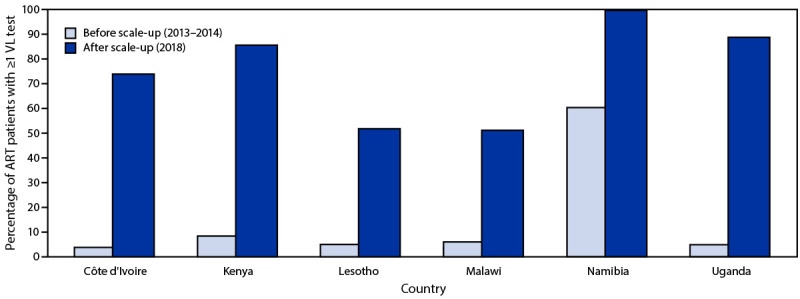
Percentage of HIV-positive patients receiving antiretroviral therapy who had ≥1 viral load test before and after scale-up of viral load testing — six sub-Saharan African countries,* 2013–2014**^†^** and 2018 **Abbreviations:** ART = antiretroviral therapy; VL = viral load. * Two countries not shown (South Africa and Tanzania) because data were only provided through June 2018. ^†^ Period before scale-up was 2014 in Côte d’Ivoire and 2013 in all other countries.

Before the scale-up, the rate of viral suppression, was ≥80% in only three of the eight countries: Uganda (90%), Malawi (86%), and Tanzania (80%) (Figure 2). By the end of 2018, all countries except Côte d’Ivoire reported viral suppression rates of ≥85%. The highest prevalence of viral suppression (94.4%) was reported by Namibia. The largest increase in viral suppression rate from 2013 to 2018 occurred in Kenya (40%), followed by Namibia (28%), and Lesotho (24%); rates increased by <20% in Côte d’Ivoire, South Africa, and Tanzania. Viral suppression rate was unchanged in Malawi, and in Uganda the rate decreased by 2.4%, while the number of viral load tests increased.

**FIGURE 2:**
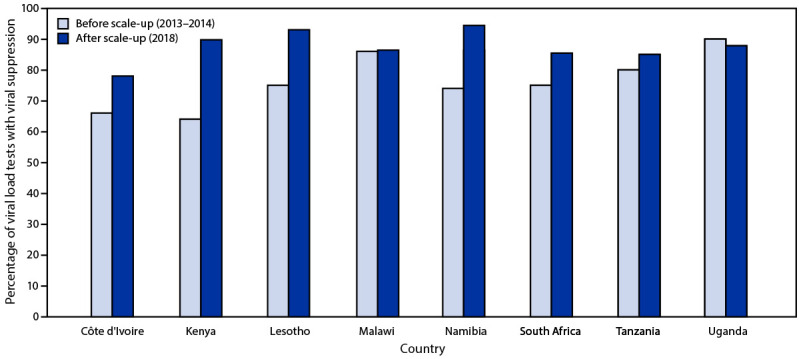
Percentage of HIV viral load tests indicating viral suppression* before and after viral load testing scale-up — eight sub-Saharan African countries,**^†^** 2013–2014**^§^** and 2018 * Viral suppression is <1,000 copies of HIV RNA per mL of blood. ^†^ Two countries not shown (South Africa and Tanzania) because data were only provided through June 2018. ^§^ Period before scale-up was 2014 in Côte d’Ivoire and 2013 in all other countries.

## Discussion

This review of scale-up of HIV viral load testing during 2013–2018 in eight sub-Saharan African countries documents successful efforts to increase access to viral load monitoring for patients receiving ART. Early in the process, many sub-Saharan African countries were just initiating viral load testing to monitor treatment success. Transitioning from using CD4 counts to molecular-based viral load testing as a national strategy required educating health care providers and patients to increase the demand for viral load testing, training laboratorians to improve the quality and efficiency of molecular testing, optimizing the laboratory network, and strengthening clinical services for effective patient management. Some of the challenges identified early in 2013 and 2014 remain, including difficulties with specimen transport, equipment breakdown, and delays in development of a skilled workforce (*8*). However, efforts by officials and health care workers to overcome these difficulties and each country’s determination to reach the UNAIDS goal of 95% of ART patients achieving viral suppression has led to continued progress in viral load monitoring. Seven of the eight countries achieved viral load suppression rates of ≥85% for all viral load tests performed during 2018; Côte d’Ivoire reported significant improvement in rates, from 53% in 2015 to 78% in 2018 (*7*).

Test result turnaround time decreased in only three countries (Kenya, Lesotho, and Uganda); turnaround time increased in four countries, highlighting the need for increased efficiency. The increased turnaround time could be explained by 1) increased testing volume and the inability of existing systems to meet this demand; 2) an increased number of facilities or service delivery points collecting specimens, leading to a more complex transport network; 3) prolonged sample storage times until pickup at facilities or hub sites; or 4) inadequate number of personnel to process the increased number of specimens at viral load laboratories. Continued capacity building is needed to address these issues.

The findings in this report are subject to at least two limitations. First, viral suppression was defined as a viral load test result of ≤1,000 HIV RNA copies per mL; prevalence cannot be determined from viral load test results for individual patients, as some data sources have patient-level duplication. Second, Malawi’s guidelines for viral load testing every 2 years were different from those in all other countries. Less frequent testing for persons in Malawi resulted in fewer viral load tests.

Effective partnerships between ministries of health and multiple international stakeholders such as PEPFAR, the Global Fund, WHO, the Clinton Health Access Initiative, the African Society for Laboratory Medicine, and others have contributed to progress in viral load monitoring. Ongoing engagement with ministries of health and finance and with officials in financial and technical areas, at national, subnational, and community levels will be required to sustain and improve current gains. Implementing best practices and data-driven program improvement strategies should assist countries to move beyond the third “95” UNAIDS goal (95% of persons on ART achieve viral suppression) to reach HIV epidemic control.

**Summary**

**What is already known about this topic?**

HIV viral load monitoring is recommended to assess antiretroviral treatment success; however, low- and middle-income countries face financial, operational, and country-specific challenges that must be overcome to adequately scale up viral load monitoring for all HIV-positive persons.

**What is added by this report?**

Sub-Saharan African countries have overcome challenges to initiate and scale up HIV viral load testing to monitor patients receiving ART. By 2018, seven of eight assessed countries reported viral load suppression rates of ≥85%. Logistical problems remain in several countries.

**What are the implications for public health practice?**

Viral load testing in low- and middle-income countries enables monitoring of viral load suppression at the individual and population level, which is necessary to achieve global epidemic control.

## Footnotes

* Deceased.

†
https://aidstargets2025.unaids.org

§
https://www.afro.who.int/health-topics/hivaids
